# Validation of an Automated AI Algorithm for the Quantification of Major OCT Parameters in Retinal Vein Occlusion–Related Macular Edema

**DOI:** 10.3390/jcm15103561

**Published:** 2026-05-07

**Authors:** Adriano Carnevali, Domenico Chisari, Raffaella Gioia, Alessandra Mancini, Massimiliano Borselli, Rosa Macrì, Andrea Lucisano, Giovanna Carnovale Scalzo, Luisa Frizziero, Vincenzo Scorcia, Edoardo Midena

**Affiliations:** 1 Department of Ophthalmology, University Magna Graecia of Catanzaro, Azienda Ospedaliera-Universitaria R. Dulbecco, 88100 Catanzaro, Italy; domenico.chisari@studenti.unicz.it (D.C.); raffaella.gioia@hotmail.it (R.G.); ale.mancini.a@gmail.com (A.M.); mborselli93@gmail.com (M.B.); rosa.macri95@gmail.com (R.M.); andrea.lucisano@unicz.it (A.L.); giovannacarnovale@virgilio.it (G.C.S.); vscorcia@unicz.it (V.S.); 2 Department of Ophthalmology, University of Padova, 35100 Padova, Italyedoardo.midena@unipd.it (E.M.); 3 IRCCS—Fondazione Bietti, 00198 Rome, Italy

**Keywords:** macular edema, retinal vein occlusion, biomarkers, optical coherence tomography, artificial intelligence, intraretinal fluid, subretinal fluid, hyperreflective retinal foci, external limiting membrane, ellipsoid zone

## Abstract

**Background/Objectives**: Retinal vein occlusion (RVO) commonly causes vision loss from macular edema (ME). OCT biomarkers (IRF, SRF, HRF, and ELM/EZ disruption) inform prognosis and treatment but are rarely quantified routinely due to time burden and interobserver variability. We aimed to validate a deep-learning algorithm for automated detection and quantification of key OCT biomarkers in RVO-ME versus expert assessment. **Methods**: In this retrospective single-center study, 93 eyes with RVO-ME imaged with spectral-domain OCT were analyzed. The AI quantified IRF/SRF volumes, ELM/EZ interruption, and HRF counts. Two masked expert clinicians provided reference evaluations. Performance and agreement were assessed using ROC AUC, Cohen’s kappa, intraclass correlation coefficient (ICC), Pearson correlation, and Bland–Altman analysis. Image-quality metrics (foveal centration and retinal layer segmentation) were recorded. **Results**: The AI showed high diagnostic performance (AUC: SRF 0.969; ELM 0.871; EZ 0.958) and substantial-to-almost-perfect agreement (kappa: SRF 0.807; ELM 0.788; EZ 0.914). HRF quantification correlated strongly with experts (r = 0.89, *p* < 0.001), with very good agreement (ICC = 0.87) and minimal bias. Image-quality accuracy exceeded 98% for foveal centration and layer segmentation. **Conclusions**: This AI software enables reliable, rapid automated assessment of major OCT biomarkers in RVO-ME, supporting streamlined personalized management; prospective studies should confirm longitudinal monitoring and treatment-guidance value.

## 1. Introduction

Retinal vein occlusion (RVO) is a potentially vision-threatening condition caused by the partial or complete obstruction of a retinal vein, and it represents the second most common major retinal vascular disorder after diabetic retinopathy [1,2]. RVO reflects the principles of Virchow’s triad, including endothelial dysfunction, venous stasis, and hypercoagulability, which may promote thrombus formation within the retinal venous circulation [1,2]. The resulting venous obstruction leads to increased intraluminal pressure, impaired retinal perfusion, tissue hypoxia/ischemia and breakdown of the blood–retinal barrier. According to the anatomical site of occlusion, RVO is commonly classified into branch retinal vein occlusion (BRVO) and central retinal vein occlusion (CRVO). BRVO typically occurs at an arteriovenous crossing, where compression of the venous wall by an adjacent arteriole may promote turbulent flow, endothelial damage, thrombosis, and downstream venous congestion. In contrast, CRVO usually occurs at or near the lamina cribrosa of the optic nerve, leading to more widespread retinal venous outflow impairment. Hemiretinal vein occlusion (HRVO) represents a form occurring at the level of the optic disk that involves venous drainage of either the superior or inferior retinal hemifield. Although BRVO is more frequent than CRVO, CRVO is generally associated with a greater risk of severe visual impairment.

The estimated global prevalence of RVO among individuals aged 30–89 years was 0.77%, corresponding to approximately 28.06 million affected people worldwide. Specifically, BRVO and CRVO accounted for prevalence rates of 0.64% and 0.13%, equivalent to 23.38 million and 4.67 million individuals, respectively. The pooled five-year and ten-year cumulative incidences of RVO were 0.86% and 1.63%, respectively. Established risk factors include advanced age, hypertension, prior myocardial infarction, stroke, elevated total cholesterol levels, and increased serum creatinine [1,2]. Given the global aging trend and the increasing prevalence of cardiovascular diseases, this number is expected to rise further, suggesting that RVO may pose a growing public health burden [1,3].

Macular edema secondary to retinal vein occlusion (RVO-ME) is the most common cause of vision loss in patients with RVO [4]. The pathogenesis of RVO-ME remains not fully understood, but contributing factors include venous obstruction leading to increased intraluminal pressure, reduced blood flow velocity, varying degrees of retinal capillary nonperfusion, retinal hypoxia, and inflammation [4]. These changes promote the upregulation of vascular endothelial growth factor (VEGF) and inflammatory mediators, causing breakdown of the blood–retinal barrier and contributing to macular edema [5].

Intravitreal anti-VEGF implants target these underlying factors and have demonstrated efficacy in preserving visual function over a period of at least two to three years [5,6].

Despite the effectiveness of anti-VEGF therapy, some patients with RVO-ME experience recurrent or persistent edema and require repeated intravitreal anti-VEGF injections [7,8]. This suggests that exclusive VEGF suppression may not fully address the complex pathophysiology of RVO-ME, which likely involves additional cytokines and inflammatory factors [9]. Consequently, there is a pressing need for alternative therapeutic strategies for patients who are resistant to anti-VEGF treatment. Corticosteroids, which modulate inflammatory pathways implicated in ME, have shown promise in this regard. As proposed in several studies on diabetic macular edema (DME), it is reasonable to hypothesize the existence of distinct RVO-ME phenotypes too, each characterized by varying disease pathogenesis, severity, progression risk and specific treatment response [4,10]. Therefore, a detailed assessment of the individual features of RVO-ME may enhance understanding of its pathophysiology and support a personalized precision approach to optimize therapeutic outcomes.

To further elucidate the pathophysiology of retinopathies, numerous studies have focused on the investigation of optical coherence tomography (OCT) “subclinical” imaging biomarkers [11,12,13]. Key OCT biomarkers—including the distribution of intraretinal fluid (IRF) and subretinal fluid (SRF), the integrity of the external limiting membrane (ELM) and ellipsoid zone (EZ), and the identification of hyperreflective retinal foci (HRF)—are increasingly recognized for their prognostic value and their role in predicting treatment response in RVO and other retinal diseases [4,12,13]. IRF, particularly when involving the ganglion cell layer (GCL), represents a negative prognostic indicator for visual acuity recovery after anti-VEGF treatment in RVO. IRF has been associated with increased CRT and poorer BCVA; in particular, GCL involvement may reflect inner retinal damage, chronicity, and ischemia, and has been linked to lower BCVA and reduced anatomical response in terms of CRT reduction [4]. In RVO, SRF is frequently observed during the acute phase. Although its mere presence does not necessarily indicate a poor prognosis or unfavorable treatment response, persistent SRF may reflect disease chronicity and a greater inflammatory component, potentially identifying cases in which intravitreal corticosteroid therapy may be beneficial [4]. In eyes with RVO-ME, preservation of ELM and EZ integrity has been associated with better visual acuity outcomes and more favorable treatment response, whereas disruption of these layers may reflect photoreceptor damage and poorer functional prognosis [4,14]. The EZ, in particular, represents a structural marker of photoreceptor outer-segment integrity and therefore provides an indirect assessment of photoreceptor health [15]. HRF are considered markers of active inflammation, as they correlate with inflammatory cytokines such as CD14, IL-1β, and IL-6, which may stimulate microglial activation [16]. Their accumulation in the outer retina has been associated with poorer visual outcomes [17]. The presence of HRF has also been associated with increased CRT, IRF, SRF, and EZ disruption [16]. Notably, corticosteroid treatment with dexamethasone implant appears to reduce HRF more effectively than anti-VEGF therapy [18]. HRF reduction following both agents has been correlated with improvement in visual acuity [16].

However, the advancement of imaging technologies has resulted in an overwhelming volume of data. The vast array of potentially relevant biomarkers, which reflect the heterogeneity of disease origins and phenotypes, presents a significant analytical challenge, rendering each patient a complex “big data” case [11,19,20,21].

The urgent need for “intelligent” tools capable of managing vast volumes of imaging data has led to the introduction of artificial intelligence (AI) in the field of medicine. AI is increasingly recognized for its role in the automated analysis of ocular imaging data across multiple retinal diseases [22]. By enabling automatic detection and quantification of key structural biomarkers, AI may improve consistency, reduce observer-related variability, and provide more objective, reproducible, and time-efficient assessments. These tools may help translate biomarker research into clinical practice by making advanced image analysis more accessible in routine settings. As AI-based systems continue to evolve and become integrated into imaging platforms, they may increasingly support personalized treatment planning and real-time clinical decision-making [22].

Despite the growing interest in OCT biomarkers and the increasing application of AI-based imaging analysis, there is still a lack of studies that simultaneously and comparatively validate automated detection and quantification of multiple key biomarkers—such as IRF, SRF, HRF, and ELM/EZ integrity—in RVO-ME. Notably, an AI software has previously shown excellent performance in the automated quantification of OCT biomarkers in DME [23,24]; however, its validation in the specific setting of RVO-ME remains limited.

In our study, we employed an AI-based tool to analyze OCT imaging biomarkers in patients with RVO-ME, with the aim of validating an easily applicable AI system for routine clinical practice. Such a tool could support personalized treatment decisions by facilitating earlier and more effective management of RVO-ME, including the timely transition to corticosteroid therapy in cases where anti-VEGF agents are insufficient. The primary outcome was the agreement between the AI-based analysis and clinical evaluation in detecting IRF, SRF, and ELM/EZ interruption, as well as in quantifying HRF. Secondary outcomes included the accuracy of OCT biomarker quantification and OCT image-quality parameters.

## 2. Materials and Methods

### 2.1. Study Design and Population

A single-center AI algorithm validation study was conducted on eyes with RVO-ME, using the same algorithm and study design as a previous successful study focused on DME (Ophthal software, v. 1.0, 20.10.2023 (Mr. Doc s.r.l., Via Enrico Fermi 43, 00146 Rome, Italy; [23]). The AI algorithm was not specifically developed for RVO-ME but was designed for the analysis of OCT biomarkers in DME [23]. However, the OCT biomarkers targeted by the software—IRF, SRF, HRF, and ELM/EZ integrity—are also commonly observed in RVO-ME and present similar structural features on OCT imaging. Therefore, the algorithm was applied in the present study to assess whether they could be accurately detected and quantified by AI in the RVO setting. The AI algorithm was pre-trained on independent datasets and was not further trained or fine-tuned on the data included in the present study.

The retrospective study adhered to the principles of the Declaration of Helsinki, and informed consent was waived by the Institutional Review Board due to complete anonymization of all imaging data. Spectral-domain OCT (SD-OCT) scans of consecutive eyes affected by RVO-ME were collected at the ‘Retina & Imaging’ center of the Ophthalmology Unit at the University of Catanzaro, “Magna Graecia”. The diagnosis of RVO-ME, including both CRVO and BRVO subtypes, was established based on clinical examination and multimodal imaging, including fundus evaluation and SD-OCT. The presence of IRF was required for inclusion; therefore, IRF was clinically present in all included eyes. For the purposes of this study, all eyes with RVO-ME were analyzed as a single cohort, without subgroup stratification.

Inclusion criteria were as follows:

- OCT scans obtained from eyes affected by RVO-ME (BRVO-ME or CRVO-ME, with IRF required for inclusion);
- Availability of both volumetric macular OCT scans and high-resolution foveal linear scans;
- OCT scans acquired using the Spectralis HRA + OCT2 platform (Heidelberg Engineering, Max-Jarecki-Strasse 8, 69115, Heidelberg, Germany) according to study acquisition protocol;
- Adequate foveal centration of the OCT scans;
- Complete visualization of all retinal layers within the acquired OCT frame, without truncation or cropping of the retinal layers;
- Absence of acquisition artifacts prevents reliable segmentation.

Exclusion criteria were as follows:

- OCT images affected by media opacities that could compromise image quality;
- Presence of any chorioretinal disease other than RVO-ME
- History of intraocular surgery or ocular conditions causing macular edema unrelated to RVO, if present.

Each scan was independently analyzed both by the automatic AI quantification software and by clinical evaluation at the study site, in order to (i) provide an initial descriptive comparison between the two approaches, (ii) assess their agreement, (iii) evaluate the accuracy of automated biomarker quantification, (iiii) evaluate the accuracy of image-quality parameters (Automated foveal center identification and retinal layer segmentation).

### 2.2. OCT Acquisition Protocol

All SD-OCT images were obtained using the Spectralis HRA + OCT2 platform (Heidelberg Engineering, Heidelberg, Germany), which provides high-resolution imaging with a nominal scanning speed of up to approximately 40,000 A-scans per second, depending on acquisition settings. For each eye, both a volumetric scan and a linear scan were analyzed. Acquisition protocol for OCT scans included a 6 × 6 mm volumetric scan composed of 49 B-scans acquired in high-resolution (HR) mode with >12 automatic real-time tracking (ART) and a quality index >28, along with a linear scan passing through the fovea acquired in HR mode with >90 ART and a quality index >30. OCT images were excluded if affected by media opacities that could compromise image quality.

### 2.3. AI Algorithm and Image Analysis

**Figure 1:**
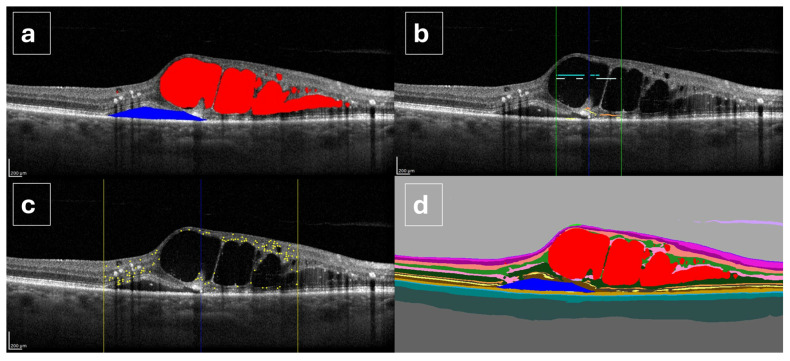
Overview of the spectral-domain optical coherence tomography biomarkers automatically analyzed by the AI software: (**a**) Intraretinal fluid (red) and subretinal fluid (blue); (**b**) External limiting membrane (orange) and ellipsoid zone (yellow) within the central 1 mm area (delimited by green lines); (**c**) Hyperreflective retinal foci (yellow dots) within the central 3 mm area (delimited by yellow lines); (**d**) Comprehensive segmentation of all retinal layers and fluid compartments.

The AI software is based on a deep learning architecture employing convolutional neural networks (CNNs) within a semi-supervised framework, combined with adversarial generative networks. The model performs automated pixel-wise segmentation of OCT images, assigning each pixel to a specific class, including intraretinal fluid, subretinal fluid, and retinal layers, thereby generating labeled segmentation masks. These segmentation outputs enable the extraction of quantitative and spatial biomarkers [25]. Specifically, the algorithm provides volumetric measurements of IRF and SRF from volumetric scans, derived through interpolation across multiple B-scans. The percentage of IRF volume was calculated within three concentric retinal regions: the central 1 mm area, the parafoveal ring between 1 and 3 mm, and the perifoveal ring between 3 and 6 mm. From the high-resolution linear scan, the number of HRF within the central 3 mm region was quantified, as described in previous studies [23]. Disruptions of the ELM and EZ are analyzed in the central 1 mm of the central B-scan passing through the fovea. The software also generates graphical outputs, including segmentation maps and labeled images highlighting individual biomarkers, such as fluid compartments, HRF, ELM/EZ interruptions, and retinal layers, alongside numerical summaries for each scan and follow-up sequence (Figure 1).

### 2.4. Clinical Evaluation and Grading

Clinical evaluation was performed by two experienced, blinded examiners (C.D. and M.R.; each with more than 7 years of experience in medical retina and OCT interpretation), who assessed the presence of IRF, SRF, and interruptions of the ELM and EZ, using a binary scale (0 = absent, 1 = present). Additionally, the accuracy of the biomarker quantification was classified as either accurate or inaccurate based on image quality. The number of HRF was manually counted for each eye by the same two blinded, trained medical retina specialists from the same reference center.

For both volumetric and linear scans, the accuracy of automated foveal centering and segmentation of retinal layers was also evaluated and recorded as accurate or inaccurate by the blinded medical retinal experts.

In case of any disagreement between the two graders, a third senior retina specialist (C.A.), professor of ophthalmology with extensive clinical and academic experience in medical retina and multimodal retinal imaging, reviewed the images and provided the final adjudication.

### 2.5. Outcomes

The primary outcome of the study was the degree of agreement between AI analysis and final clinical evaluation for detecting IRF, SRF, ELM/EZ interruptions and HRF count. Secondary outcomes included the accuracy of biomarker quantification and assessment of OCT scan quality parameters (Foveal Centration and Retinal Later Segmentation).

### 2.6. Statistical Analysis

All statistical analyses were performed using SPSS Statistics, version 31 (IBM Corporation, Armonk, NY, USA).

The parameters analyzed in the present study included IRF, SRF, ELM integrity, EZ integrity, and HRF. These parameters were summarized using standard descriptive statistics: means and standard deviations for quantitative variables, and absolute frequencies with corresponding percentages for qualitative variables.

The validation process consisted of comparing the results provided by the AI-based system with those from clinical evaluations.

Receiver operating characteristic (ROC) curves were generated for SRF, ELM, and EZ, using the clinical assessment as the reference standard. Diagnostic performance was expressed with the area under the ROC curve (AROC). For SRF, an optimal cutoff was defined by considering multiple criteria: Youden’s index, Euclidean distance from the ideal point (0,1), and the difference between sensitivity and specificity.

The agreement between the AI system and clinical evaluation was assessed using Cohen’s kappa coefficient. Interpretation followed conventional thresholds: 0 indicates no agreement; 0.01–0.20 slight; 0.21–0.40 fair; 0.41–0.60 moderate; 0.61–0.80 substantial; and 0.81–1.00 almost perfect agreement.

Agreement for HRF count, being a continuous variable, was studied using the Bland–Altman method. Additionally, the intraclass correlation coefficient (ICC) with its 95% confidence interval was calculated to assess measurement consistency. A scatter plot was generated to visually assess the relationship between HRF values measured manually and those obtained via AI. The degree of correlation was quantified using Pearson’s correlation coefficient.

## 3. Results

### 3.1. Study Population

A total of 93 eyes with RVO-ME were included in the analysis. According to the definition of macular edema, IRF was detected in all cases by the AI software. The mean IRF volume, as measured by the software, was 0.765 ± 1.160 mm^3^ (range: 0.001–7.009 mm^3^). The distribution of IRF within the macular subfields was as follows: 24.06 ± 23.55% in the central 1 mm circle, 42.05 ± 20.06% in the 3 mm ring, and 33.88 ± 27.91% in the 6 mm ring. SRF was identified by the AI software when its estimated likelihood exceeded a predefined threshold (≥0.002 mm^3^; sensitivity: 89.4%, specificity: 96.0%, Youden index: 0.897). Based on this criterion, SRF was detected in 18 eyes, with a mean volume of 0.040 ± 0.200 mm^3^ (range: 0.002–1.630 mm^3^). Disruptions of the ELM and/or the EZ were detected in 23 eyes (24.47%) and 47 eyes (50.00%), respectively. In the central 1 mm area, the mean percentage of interruption was 8.92 ± 20.74% for the ELM and 36.27 ± 44.09% for the EZ. The mean number of HRF automatically counted by the AI software within the central 3 mm of the high-resolution linear scan was 103.63 ± 43.38. The main clinical and morphological characteristics of the study population are summarized in Table 1.

### 3.2. Descriptive Comparison Between AI-Based Analysis and Clinical Evaluation

Table 2 summarizes the comparison between AI-based analysis and clinical evaluation for the main OCT biomarkers. The distribution of categorical biomarker detection was comparable between the two approaches, with no statistically significant differences observed for SRF, ELM, and EZ detection (*p* > 0.05 for all comparisons). Similarly, HRF counts did not differ significantly between AI-based and manual assessment (103.6 ± 43.4 vs. 101.7 ± 35.6; *p* = 0.702).

**Figure 2:**
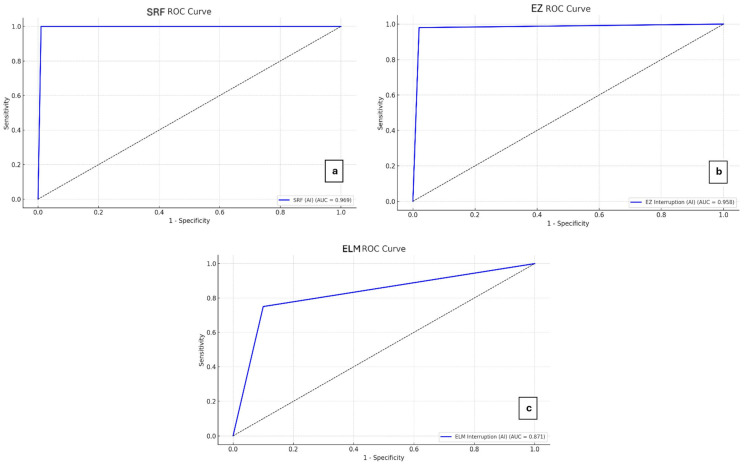
Receiver operating characteristic (ROC) curves illustrating the diagnostic performance of the artificial intelligence (AI) software in comparison with manual clinical evaluation for the following retinal biomarkers: (**a**) subretinal fluid (SRF), (**b**) ellipsoid zone (EZ), (**c**) external limiting membrane (ELM).

AROC values reflecting the diagnostic performance of the AI system compared to manual clinical evaluation were 0.969 for SRF, 0.871 for ELM, and 0.958 for EZ (Figure 2). According to standard interpretation thresholds, the AI system showed excellent diagnostic performance for SRF and EZ detection (AUC > 0.9) and good performance for ELM (0.8 < AUC < 0.9).

### 3.3. Agreement Between AI-Based Analysis and Clinical Evaluation

The percentage of agreement between the AI software and clinical evaluation was 94.62% for SRF volume, 95.70% for EZ integrity, and 91.40% for ELM integrity. Inter-rater agreement assessed by Cohen’s kappa (95% confidence interval) between AI software and clinical evaluation was 0.807 (±0.082 S.E.) for SRF volume, 0.788 (±0.071 S.E.) for ELM integrity, and 0.914 (±0.042 S.E.) for EZ integrity. According to standard interpretation thresholds, agreement between AI and clinical evaluation was almost perfect for SRF and EZ (κ > 0.80), and substantial for ELM (0.61 < κ < 0.80).

**Figure 3:**
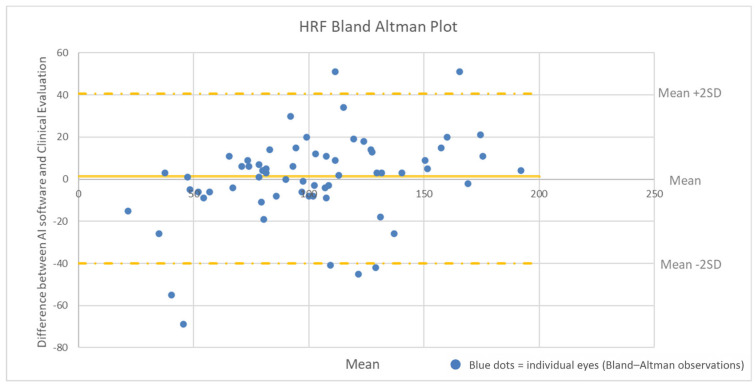
Bland–Altman plot analysis comparing the number of hyperreflective foci (HRF) assessed by artificial intelligence (AI) software and clinical evaluation. AI: artificial intelligence; SD: standard deviation.

Agreement between AI and clinical evaluation for HRF count was assessed using Bland–Altman plot analysis (Figure 3). Nearly all values fell within the ±2 standard deviation (SD) range. The mean difference between AI and manual counts was 1.194 ± 19.769, with no evident systematic trend. The ICC was 0.87 (95% confidence interval: 0.83–0.91), indicating very good concordance.

**Figure 4:**
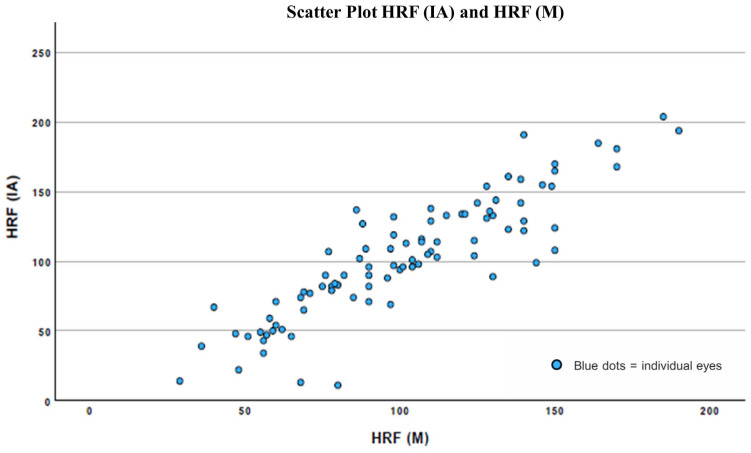
Scatter plot comparing the number of hyperreflective foci (HRF) detected by the artificial intelligence (AI) system [HRF (IA)] and by manual clinical evaluation [HRF (M)]. Each blue dot represents a single eye. A strong positive linear correlation was observed (Pearson’s r = 0.89, *p* < 0.001), supporting the significant correlation between the two quantification methods.

Scatter plot analysis revealed Pearson’s correlation coefficient of 0.89 (*p* < 0.001) between HRF values obtained by the AI-based system and manual clinical evaluation (Figure 4).

### 3.4. Accuracy of Automated Biomarker Quantification

Qualitative clinical evaluation confirmed the accuracy of the automatic quantification in 89 eyes (95.70%) for IRF and 85 eyes (91.40%) for SRF. Regarding ELM and EZ integrity, the AI-based analysis was clinically consistent with manual assessment in 84 eyes (90.32%) for ELM interruption and in 86 eyes (92.47%) for EZ interruption.

### 3.5. Accuracy of OCT Image-Quality Parameters (Automated Foveal Center Identification and Retinal Layer Segmentation)

In terms of foveal center identification, an inaccurate result was observed in two eyes on the map scan and in none on the linear scan. Automated retinal layer segmentation was considered clinically inaccurate in only one eye on the map and in 0 eyes on the linear scan out of the total 93 eyes evaluated.

## 4. Discussion

In this study, we validated an AI-based algorithm for the automated analysis of OCT biomarkers in eyes with RVO-ME, demonstrating high concordance with expert clinical evaluation. In line with the primary outcome, the AI system showed almost perfect agreement with clinicians for SRF and EZ detection (κ = 0.807 and κ = 0.914, respectively), and substantial agreement for ELM disruption (κ = 0.788). HRF quantification also showed strong concordance with manual assessment, with a Pearson correlation coefficient of 0.89 and an ICC of 0.87. According to recent classification systems, the HRF quantified by our AI algorithm corresponds to neuro-inflammatory HRF—small (<30 μm), of intermediate reflectivity, lacking posterior shadowing, and occurring throughout the retinal layers [26]. The relatively wide limits of agreement observed in the Bland–Altman analysis suggest that AI-derived HRF counts may have some degree of variability at the individual-eye level and should be interpreted as supportive quantitative indicators rather than as perfectly interchangeable with manual measurements in every single case. However, the absence of a systematic bias and the overall consistency of the results support the potential utility of AI for standardized assessment of HRF Regarding secondary outcomes, the algorithm demonstrated high accuracy in biomarker quantification, with clinically consistent results in 89 eyes (95.70%) for IRF, 85 eyes (91.40%) for SRF, 84 eyes (90.32%) for ELM, and 86 eyes (92.47%) for EZ. OCT image-quality parameters were also reliable, with inaccurate foveal centration observed in only two eyes on volumetric scans and inaccurate retinal layer segmentation in one eye.

Despite the significant technological advancements and the demonstrated efficacy of intravitreal therapies in clinical trials, real-world therapeutic outcomes of the most frequent retinal diseases have failed to match expectations. Numerous studies have highlighted the difficulty of reproducing trial-level results in real-world clinical settings, largely due to delays in detecting disease onset, recurrence, or progression—factors that are critical for timely therapeutic intervention. These delays contribute to a reduction in patients’ long-term visual function and quality of life and impose substantial financial burdens on healthcare systems [27,28,29,30,31].

In an effort to identify new indicators of disease progression and therapeutic response, different OCT imaging biomarkers with promising predictive and prognostic potential have been described in RVO and in other major maculopathies: key RVO-biomarkers include CRT, HRF, SRF, IRF, and the integrity of ELM and EZ, among others [4,32].

However, the high volume of data generated by each scan and the subjective quantification of OCT biomarkers make the evaluation of OCT biomarkers excessively time-consuming and highly prone to human error. This makes their evaluation on a regular basis in a clinical setting currently unfeasible, despite their potential utility.

In this context, AI may represent a practical solution to overcome the current limitations of OCT biomarker evaluation in routine clinical practice. By enabling automated, rapid, and reproducible quantification of key structural features, AI has the potential to make biomarker-based evaluation more feasible in daily clinical workflows. In a real-world scenario, this may provide clinicians with a broader and more objective view of disease course, allowing earlier identification of disease progression, recurrence, or insufficient treatment response. These technologies are bridging the gap between research findings and clinical applicability, making advanced biomarker analysis more accessible in routine practice. Moreover, the integrated assessment of multiple OCT biomarkers may help identify distinct disease phenotypes, with potential implications for treatment selection and individualized management strategies, as already suggested by recent AI-assisted studies in DME [24].

In line with these considerations, our results support the potential clinical applicability of AI-based OCT analysis in RVO-ME. Following the image upload, the software rapidly identified the fovea and simultaneously quantified all the principal biomarkers within seconds. The AI system demonstrated a high level of agreement with expert clinical evaluation across the main OCT biomarkers, indicating that AI-based tools may represent valuable support in routine practice, enabling a more consistent and scalable evaluation of disease activity. Therefore, the validation of this algorithm in the RVO setting may represent a step toward the integration of automated biomarker analysis into real-world clinical workflows, with potential benefits in monitoring disease progression and optimizing treatment strategies.

Nonetheless, this study has several limitations. First, this was a retrospective, single-center study with a relatively modest sample size. Second, no longitudinal follow-up was available; therefore, the ability of the AI system to monitor biomarker changes over time could not be assessed. Although the AI tool is capable of analyzing follow-up scans and tracking changes in individual biomarkers, future studies are needed to validate its longitudinal performance in RVO-ME. Third, no direct correlation between AI-derived metrics and functional outcomes was performed, limiting the possibility of determining the prognostic significance of automated biomarker quantification. Moreover, although image-quality parameters showed high overall reliability, a small number of errors in automated foveal centration and retinal layer segmentation were observed. Finally, all OCT scans were acquired using a single SD-OCT platform. Future multicenter, longitudinal studies should evaluate the ability of AI-based biomarker quantification to predict disease progression, treatment response, and long-term functional outcomes in real-world RVO-ME populations.

## 5. Conclusions

This study suggests that an AI-based system can reliably identify and quantify key OCT biomarkers in eyes with RVO-ME, showing a high level of concordance with expert clinical evaluation. These findings support the potential integration of automated OCT analysis into routine clinical practice, where rapid and reproducible biomarker assessment may enhance disease monitoring and facilitate earlier identification of progression or suboptimal treatment response. In this context, AI may represent a practical tool to support clinical decision-making and promote a more standardized and objective approach to patient management in real-world settings. Further large-scale, real-world studies are required to validate the software’s capability to track biomarker changes over time and to clarify the clinical significance of these changes in relation to disease progression and patient outcomes.

## Figures and Tables

**Table 1:** Clinical and morphological characteristics of the whole population.

Parameter	Value
Eyes, *n*	93
Age, years, mean ± SD	66.2 ± 11.6
Type of RVO, *n* (%)	
BRVO	73 (78.5%)
CRVO	20 (21.5%)
IRF, mm^3^, mean ± SD (Range)	0.765 ± 1.160 (0.001–7.009)
IRF distribution, %, mean ± SD	
0–1	24.06 ± 23.55
1–3	42.05 ± 20.06
3–6	33.88 ± 27.91
SRF, mm^3^, mean ± SD (Range)	0.040 ± 0.200 (0.002–1.630)
ELM, %, mean ± SD (Range)	8.92 ± 20.74 (0–85)
EZ, %, mean ± SD (Range)	36.27 ± 44.09 (0–100)
HRF, *n*, mean ± SD (Range)	103.63 ± 43.38 (11-204)
Q index, mean ± SD	31.2 ± 4.9

SD: standard deviation; RVO: retinal vein occlusion; BRVO: branch retinal vein occlusion; CRVO: central retinal vein occlusion; IRF: intraretinal fluid; SRF: subretinal fluid; ELM: external limiting membrane; EZ: ellipsoid zone; HRF: hyperreflective retinal foci.

**Table 2:** Comparison between AI-based analysis and clinical evaluation of SD-OCT biomarkers.

	AI Algorithm	Clinicians	*p* Value (a)
IRF, mm^3^	Mean ± SD	N.A.	N.A.
	0.765 ± 1.160		
	Range		
	0.001–7.009		
SRF *, *n* (%)	Absent	Absent	0.432
	75 (80.7)	80 (86)	
	Present	Present	
	18 (19.3)	13 (14.0)	
ELM interruption, *n* (%)	Absent	Absent	0.414
	70 (75.3)	64 (68.8)	
	Present	Present	
	23 (24.7)	29 (31.2)	
EZ interruption, *n* (%)	Absent	Absent	0.883
	46 (49.5)	48 (51.6)	
	Present	Present	
	47 (50.5)	45 (48.4)	
HRF, *n*	Mean ± SD	Mean ± SD	0.702 ^b^
	103.6 ± 43.4	101.7 ± 35.6	
	Range	Range	
	11.0–204.0	29.0–190.0	

a Fisher exact test. ^b^ Independent sample Student *t*-test. * Cut-off value: 0.002 mm^3^. AI: artificial intelligence; IRF: intraretinal fluid; SRF: subretinal fluid; ELM: external limiting membrane; EZ: ellipsoid zone; HRF: hyperreflective foci; SD: standard deviation; *n*: number; N.A.: not applicable.

## Data Availability

The data that support the findings of this study are available from the corresponding author upon reasonable request.

## Abbreviations

The following abbreviations are used in this manuscript:

RVO	Retinal Vein Occlusion
ME	Macular Edema
RVO-ME	Macular Edema secondary to Retinal Vein Occlusion
VEGF	Vascular Endothelial Growth Factor
DME	Diabetic Macular Edema
OCT	Optical Coherence Tomography
IRF	Intraretinal Fluid
SRF	Subretinal Fluid
ELM	External Limiting Membrane
EZ	Ellipsoid Zone
HRF	Hyperreflective Retinal Foci
AI	Artificial Intelligence
SD-OCT	Spectral-domain OCT
HR	High-Resolution
ART	Automatic Real-Time Tracking
DL	Deep Learning
ROC	Receiver operating characteristic
AROC	Area under the ROC curve
ICC	Intraclass Correlation Coefficient
SD	Standard Deviation
nAMD	Neovascular Age-Related Macular Degeneration
